# Psychedelics: Safety and Efficacy

**DOI:** 10.3390/ijerph22020134

**Published:** 2025-01-21

**Authors:** Norman Miller

**Affiliations:** Medical College of Georgia, Augusta University, Augusta, GA 30912, USA; milleradvocatespllc@outlook.com

**Keywords:** psychedelics, efficacy, addiction, toxicity, safety

## Abstract

Psychedelic research has experienced a renaissance in recent years, with many researchers exploring the possible therapeutic effects of these drugs. Medical institutions, universities, pharmaceutical companies, and governmental institutions alike have been showing an increase in support of this research, as shown through the recent establishment of various psychedelic research facilities across the United States. However, the safety and efficacy of psychedelic usage are not the focus of the existing research. Additionally, many of the studies on psychedelic use that have already been published lack the necessary scientific rigor required for studies of such medical importance. The current paper will discuss the issue of misleading information as well as poorly designed studies and what implications these have on the rescheduling of psychedelic drugs that are currently categorized as Schedule I. The current Schedule I status of psychedelics means that they are considered to have no legitimate medical value as well as a high potential for abuse. Furthermore, the current paper aims to discuss psychedelics while drawing awareness to the lack of the literature surrounding the safety and efficacy of psychedelics for medical use. This paper will also discuss the history of psychedelic use and abuse, the adverse effects of psychedelic use, and the role that personal and financial bias has within the psychedelic research field.

## 1. Introduction

As more and more states legalize marijuana, political officials and the general public alike are taking an interest in the movement toward legalized psychedelics and their medical usage. Psychedelic drugs (or “classic psychedelics”) “mainly interact with specific receptors, which are molecular structures in the brain” [1]. These psychedelics may “bring on vivid visions or sensations, alter a person’s sense of self, and promote feelings of insightfulness or correction” [1]. While dissociative drugs (such as ketamine) may also alter perception, they are distinguished by their ability to “make people feel disconnected from their body and environment” [1]. “Effects of psychedelic and dissociative drugs may be difficult to predict and depend on many factors” [1]. Some adverse effects of psychedelic use include extreme emotions (such as fear, confusion, or panic), hallucinations, nausea, changes in heart rate, and “impaired thought processes and perception” that may lead individuals to “behave in unusual and sometimes dangerous ways” [1]. In brief, psychedelic drugs produce hallucinatory and out-of-body experiences. Further, the DSM-5 outlines various disorders related to addiction to psychedelic substances [2].

Many psychedelic drugs are alkaloids. Indole alkaloids (derived from the amino acid tryptophan) include psilocybin, dimethyltryptamine, and ibogaine. While indoleamines can be divided into two subgroups (tryptamines and ergolines), most psychedelics are derived from tryptophan. Other psychedelics may fall under a different amino acid category. As an example, mescaline (found in peyote cactus) is categorized within phenethylamines, whereas lysergic acid diethylamide (LSD) is categorized within ergolines.

The popular claim among proponents of psychedelics is that they can be used to treat mental conditions such as post-traumatic stress disorder (PTSD), anxiety, and depression [3,4]. However, the literature surrounding the use of psychedelics is strongly lacking in information regarding the drugs’ adverse effects, efficacy, and overall safety for medicinal use [5,6]. Visual and auditory hallucinations (and dissociation) are typically considered to be adverse effects [1]. However, psychedelic researchers argue that these effects may allow an individual under the influence of these drugs to be more susceptible to therapeutic practices. Many claim that this gap in the literature is due to governmental involvement during the 1970s and 1980s and that it was especially difficult to conduct research on psychedelics after the implementation of the 1970 Controlled Substance Act [7,8]. While current research is attempting to fill this gap, the amount of information currently available is not nearly enough to consider psychedelics safe for therapeutic use.

### 1.1. Current Schedule I Status of Psychedelics and CSA Requirements for Rescheduling

The Controlled Substance Act was established with the purpose of preventing diversion and misuse of controlled substances [9]. The creation of the Controlled Substance Act was primarily a result of the cultural and political culture at the time, though it also involved two international treaties: The Single Convention on Narcotic Drugs of 1961 and The Convention on Psychotropic Substances of 1971 [10,11]. The Controlled Substance Act consists of various schedules, which categorize drugs by their accepted medical use, potential abuse/addiction, and harmfulness [12].

The Controlled Substance Act categorized psychedelics as Schedule I drugs, meaning that they currently have no accepted medical use as well as a high potential for abuse and addiction [13]. However, due to their illegal status, the National Institute on Drug Abuse (NIDA) has created a process through which researchers can obtain the drug legally for research purposes. Before requesting psychedelics from NIDA for their studies, researchers must first submit a “request package” which contains a cover letter, the researcher’s curriculum vitae, the research protocols (including study goals, number of participants, and dosage of substances), and several other items [14]. Not only does NIDA give substances to research trials in the United States, but other countries also depend on them for sourcing substances. Additionally, NIDA itself has conducted research on the safety and efficacy of psychedelics, though minimal information has been published as of current [1].

To reschedule a drug for prescribing purposes (or determine which schedule a drug belongs in), the Controlled Substance Act (CSA) outlines specific factors that must be satisfied [12]. These factors are as follows: the drug’s actual or relative potential for abuse/addiction; scientific evidence of the drug’s pharmacological effect (if known); the state of current scientific knowledge regarding the drug or other substance, the drug’s history and current pattern of abuse, the scope, duration, and significance of abuse, what (if any) risk there is to the public health, the drug’s psychic or physiological dependence liability, and whether the substance is an immediate precursor of a substance already controlled under this subchapter [12]. These factors must be considered by current psychedelic research when attempting to establish a potential therapeutic use of psychedelics. Without the consideration of these factors, psychedelics cannot be considered safe for public medicinal or therapeutic use.

### 1.2. Personal Bias Within Psychedelic Research

There are a multitude of reasons leading to a significant underreporting of adverse effects and the safety of psychedelic use within the medical research literature; however, one of the most concerning potential reasons is bias. It is not unreasonable in any way for researchers to be passionate and excited about their work; however, personal bias is a large issue when it comes to reporting scientific information. Manoj Doss, a cognitive neuropsychopharmacologist and post-doctoral research fellow at Johns Hopkins University, was interviewed by “*The Microdose*” (a journal dedicated to providing updates and information within the field of psychedelics) and asked, “Beyond combining expertise from these various brain science disciplines, what other challenges are there in carrying out solid research on the neuroscience of psychedelics?” [15].

Doss, in turn, illustrated the prevalence of previous psychedelic use by researchers. Doss states that he knows “only one psychedelic researcher who’s never done psychedelics” and that he believes “this means you’ve got some stakes in the game: you did drugs, you liked them, now you’re studying them. You’re going to be tempted to say mostly positive things about them. There’s a conflict of interest” [15]. That being said, researchers within this field may also find their objectivity reduced due to other pressures, such as “the need to commit themselves wholeheartedly (or single-mindedly) to a scientific research paradigm to successfully publish and to win grants” as well as even “the unconscious need to protect their egos from injury when they have devoted their lives to a certain scientific program” [16].

It is also not uncommon for participants in these psychedelic studies to have previously used psychedelic drugs [6]. Apart from the illicit status of the drug, previous use may affect the outcome of these studies. As demonstrated by Doss, those who have a vested interest in the drug will be inclined to supply a positive report [15]. This, in turn, may harm the validity of their study. It is necessary to examine the effects of psychedelics on participants who have no history of previous use.

### 1.3. Pharmaceutical Involvement Affecting Psychedelic Research

Personal bias resulting from previous psychedelic usage is not the leading factor in the potential misrepresentation of information regarding psychedelics. Rather, it is financial pressure and biases that arise as a result. Pharmaceutical companies have a vested interest in the introduction of psychedelics to the medical market (largely due to financial gain). As demonstrated during the opioid crisis, pharmaceutical companies are financially driven and are willing to invest their money into any drug that can potentially boost revenue (whether it has positive or negative effects on an individual’s health) [17,18].

However, to introduce a drug to the medical market, there must be a significant amount of research demonstrating its medical benefits [19]. Because of this, pharmaceutical companies allocate funds to researchers, universities, and private institutions via grants [20,21,22,23]. Pharmaceutical companies, however, are primarily focused on the financial benefits that legalizing these drugs may provide. This has been consistently shown through examples of “producing false research findings, ghost writing journal articles, marketing drugs for uses that had not been previously approved by the U.S. Food and Drug Administration (FDA)” and providing “kickbacks and bribes to doctors in exchange for drug prescriptions” [24]. Pharmaceutical companies continually prove that they will put financial gains over the interest of public health, whether it is achieved by “marketing drugs in a variety of unethical ways intended to increase sales including falsifying research findings, deceptively advertising drugs”, or “paying physicians to write prescriptions” [24,25,26].

The present review primarily focuses on the safety and efficacy of psychedelics, as well as the absence of the medical literature on the topic. While psychedelic research makes optimistic claims, there is a severe underreporting of adverse effects. Due to this, and in addition to poorly constructed studies, psychedelics can not currently be considered safe for medicinal use. It is necessary that future research undergo the necessary steps to conduct reliable studies on the short and long-term safety of psychedelic use before making claims on the efficacy of the drugs. Additionally, it is imperative that researchers publish their null hypotheses in addition to all adverse effects that occur during the span of this study. Without honest and transparent research, the safety of psychedelic use will remain questionable at best.

## 2. Brief History of Psychedelics

### 2.1. Early Uses of Psychedelics

Psychedelic drugs have recorded usages within a variety of eras and ancient cultures [27]. Carod-Artal (2015) writes that the “Olmec, Zapotec, Maya and Aztec used peyote, hallucinogenic mushrooms, and the seeds of ololiuqui, that contain mescaline, psilocybin and lysergic acid amide, respectively” [28]. Aztec shamans reportedly used psilocybin to treat toothaches and stomach pain, though psychedelics were later reported to be used to treat anxiety and rheumatism [29]. These same psychedelics were also used by the Aztec people for religious purposes such as ceremonies [29]. Additionally, the Maya reportedly drank a concoction called balché, a drink made of honey and extracts of Lonchocarpus [30]. Balché was consumed in order to achieve a state of intoxication for group ceremonies [30]. Furthermore, some Native American tribes reportedly used peyote as long ago as 5700 B.C., as discovered through archeological evidence [31]. Van Court et al. (2022) explain that the earliest concrete evidence of psychedelic use for ritual ceremonies was “recorded in the Codex “Yuta Tnoho” or “Vindobonensis Mexicanus I” in Mesoamerica [29]. This codex dates back to the early 1500s CE and establishes the historical prevalence of psychedelics as well as their cultural and medicinal purpose within these cultures.

### 2.2. LSD and the Hippie Counterculture

Lysergic acid diethylamide (LSD) was not invented until 1943. It was discovered by Albert Hofmann, who was also the first person to ingest the drug [32]. The term psychedelics was later established in 1957 by Humphry Osmond [33]. Humphry Osmand, who was at the time working at the Saskatchewan Provincial Mental Hospital, created the term to reflect his own experience with the drug when trying to understand schizophrenia [33]. In the early 1960s, LSD usage became much more prevalent. Its use was especially popularized within the “hippie” counterculture movement. This can be seen largely within the “Summer of Love” social phenomenon, which encompassed thousands of young people in San Francisco. These psychedelics (but especially LSD) were used in an attempt to achieve an almost spiritual experience or as “liberating agents” [34]. The rise of hippie counterculture in the 1960s saw rampant, excessive, and overall highly dangerous and out-of-control use of psychedelics, leading to widespread concern about the safety of psychedelic drug use.

There were several recorded cases of individuals having out-of-control psychotic and often violent episodes, in addition to other notable mental health issues, including severe anxiety, high blood pressure, abdominal pain, and vomiting [7]. In the interest of public health, psychedelic drugs and their effects were examined scientifically and were considered unsafe for use within the United States. After the passing of the Controlled Substance Act in 1970, psychedelics were classified as Schedule I drugs, meaning that they had no valid medical use accompanied by a high potential for abuse [13]. Due to the Schedule I status of psychedelics, research regarding psychedelic drugs and their effects has only recently been able to continue.

## 3. Early Psychedelic Research

Before the establishment of the Controlled Substance Act, several psychedelic-centered studies were completed in order to study both the medical and spiritual effects of psychedelics. These studies were widely varied, including an emphasis on disciplines such as psychology, psychiatry, neuro-science, anthropology, sociology, as well as religious studies [35]. Due to the wide variety of research, psychological effects, and therapeutic implications ranged greatly between clinical reports. Despite such a large amount of varied research disciplines, information regarding the safety and efficacy of psychedelic use is still severely limited. This is likely due to the manner in which early research studies were designed. The adverse effects of psychedelic usage are often underreported due to improper study design (a lack of systematic assessment), poor sample selection, or to hide adverse effects [36]. A thorough understanding of adverse effects requires great attention to detail and transparent disclosure when reporting, in addition to structured, methodical testing [36]. Thus, without scientifically rigorous and detailed research on the topic of the safety and efficacy of psychedelics, it is impossible to consider them safe and effective for medical use.

### The Good Friday Experiment

One often cited early study on psychedelic use was the Good Friday Experiment (also known as the Marsh Chapel Experiment), conducted by Walter Pahnke in 1962. This study is considered especially noteworthy as it was one of the first to examine psychedelics and their potential link to spirituality or mystical experiences. Through this study, Pahnke tested his theory that psychedelic drugs (specifically psilocybin in this instance) could “facilitate a “mystical” experience in religiously inclined volunteers who took the drug in a religious setting” [37]. Pahnke enlisted a sample of twenty divinity students and randomly divided them into two groups for a double-blind experiment. Within this experiment, half of the volunteers received psilocybin, while the other half (the control group) were given niacin as an active placebo. Pahnke believed that the best environment for his study would be somewhere familiar (a religious environment specifically) where volunteers would be comfortable. Thus, he chose to conduct his experiment during a Good Friday service at the Marsh Chapel at Boston University.

Concluding this study, eight students who had received psilocybin reported a “mystical experience”, with only one student within the control group reporting feeling a sense of “sacredness and peace” [38]. In a follow-up to this experiment (25 years later), a majority of volunteers who had received psilocybin reported the experience as “one of the high points of their spiritual life”. Due to this, this study was largely considered a “success”. However, there are several large issues to note within this study. This study was intended to be a double-blind experiment; however, it was not difficult to tell which students had received the psilocybin and which had received the placebo due to alleged outbursts and actions. As an example, individuals who had received the placebo reportedly sat calmly while those using psychedelic drugs were stumbling about the room, proclaiming “God is everywhere”, in addition to other similar statements [38].

Rick Doblin, a psychedelic researcher, published a follow-up and methodological critique of Pahnke’s Good Friday Experiment in which he discussed potential issues within this study [37]. Notably, Doblin critiqued Pahnke’s poorly designed double-blind procedure. Doblin went on to discuss several other issues, including flawed questions within Pahnke’s questionnaire and a blatant lack of reporting on Pahnke’s part, specifically regarding adverse effects. Pahnke left out a considerably important event that occurred during his study. Several volunteers within his study experienced severe anxiety after being administered psilocybin. One of these volunteers had to be physically restrained at one point in the experiment. The volunteer in question was injected with Thorazine after his outburst” [39]. Doblin, unsurprisingly, felt that Pahnke’s study led to “a considerable doubt on the assertion that mystical experiences catalyzed by drugs are in any way inferior to non-drug mystical experiences in both their immediate content and long-term effects” [37].

## 4. Adverse Effects of Psychedelic Use

While popular, the use of psychedelics as a therapeutic treatment is still an emerging theory. There are many unknown factors when it comes to the overall safety and efficacy of the drugs. Additionally, there is still a lack of information on both the short and long-term effects of psychedelic use. Some currently known side effects are headache, abdominal pain, nausea or vomiting, high blood pressure, rapid heartbeat, trembling, diarrhea, fear, and anxiety [1]. Fear and anxiety typically occur during the course of a “bad trip”, though it is currently unknown how common or severe these experiences may be [1]. Additionally, psychedelic use has been linked to “dangerous behavior and injuries” [1]. Psychedelic use leads to impaired judgment and mental function, explaining why psychedelic use may cause abnormal and potentially aggressive behavior. NIDA has cited a survey of almost 2,000 individuals who have used psychedelics recreationally and had a “challenging experience” [1]. Out of these individuals, 11% reported that they had “put themselves or others at risk of harm” [1,40].

Further research is necessary to understand the extent to which these experiences may occur. It is important to realize that these effects vary widely between individuals who use psychedelic drugs. In fact, there is a large variety of factors that may impact an individual’s experience with psychedelics. Some of these factors include (but are not limited to) an individual’s “age, unique biology, sex, personality, history of drug use, mood, expectations, mindset, and surroundings” [1]. Research must account for all of these factors to truly understand the potential effects of psychedelic use before it can be considered safe for medicinal use.

### 4.1. Addiction Potential of Psychedelic Drugs

Some research suggests that addiction is not a potential outcome of psychedelic use, though this research is very limited. Some researchers assert that a large reason why psychedelics are not addictive is due to the negative side effects that accompany the use of these drugs [41,42]. They suggest that adverse effects (such as headaches or nausea) deter an individual from further psychedelic use, therefore hindering the potential for abuse [43]. This has also been supported by the low mortality rate from psychedelic use. According to the 2021 National Survey on Drug Use and Health, among people aged 12 or older in 2020, only 0.2% (about 493,000 people) had a hallucinogen use disorder within the past 12 months [43]. It is possible that a lack of reporting has an effect on the present data. While these data appear promising, it is necessary to note that “among young adults aged 19 to 30 in 2021, an estimated 8% reported using any hallucinogen, representing an all-time high since the category was first surveyed in 1988” [44].

This rise in psychedelic use may be attributed to an increased tolerance of the drugs. Increased tolerance from psychedelic use develops rapidly, resulting in an individual needing to ingest increasingly higher amounts of the drug to achieve the desired effects [45]. However, tolerance alone is not indicative of addiction. Rather, addiction is characterized by a loss of control of use. This loss of control of use (primarily seen as increased and erratic use) is often accompanied by an increased tolerance, cravings, and withdrawal symptoms from regular use.

Addiction to psychedelics (particularly ketamine) gained media attention rapidly following the death of actor Matthew Perry [46]. Perry reportedly died after overdosing on ketamine that was obtained through illegal means [46]. One doctor involved in Perry’s overdose is being “charged with seven counts of distribution of ketamine and two counts of altering and falsifying documents or records related to the federal investigation” [46]. Perry himself has been open regarding his history of addiction, even discussing it in his memoir [47]. Prior to his death, he had allegedly been “undergoing ketamine infusion therapy for depression and anxiety” [48]. While this process was supposedly monitored by medical professionals, he later sought out “ketamine vials and lozenges in the fall of 2023 outside of supervised settings until his ketamine addiction was spiraling out of control” [48]. Perry’s continuation of use despite adverse effects can be categorized as addiction. This is additionally supported by his prior history of addiction.

Ketamine has been used at parties, raves, and clubs due to its hallucinatory effects [49]. When an overdose of ketamine occurs, individuals may experience amnesia, seizures, dangerously slowed breathing, or other serious adverse effects [50]. While Perry’s case is concerning, it is not the first to occur in this manner. Ketamine rose in popularity (especially in areas such as Hollywood) after the general public became concerned with the dangerous effects of cocaine [51]. Perry’s overdose illustrates the addictive potential of ketamine and other psychedelic drugs, calling into question the safety and efficacy of the drug should it be approved for therapeutic use.

### 4.2. DSM-5: Psychedelic-Related Disorders

The DSM-5 contains clear descriptions and requirements for the diagnosis of phencyclidine use disorder (PCP use disorder) in addition to hallucinogen use disorder [2]. PCP use disorder includes all phencyclidine-like substances such as PCP, ketamine, cyclohexylamine, and dizocilpine [2]. Additionally, the DSM-5 has conducted independent field trials to ensure diagnosis reliability [2]. The DSM-5 defines PCP use disorder as a “pattern of phencyclidine (or a pharmacologically similar substance) use leading to clinically significant impairment or distress, as manifested by at least two of the following, occurring within a 12-month period” [2]. Some of the following symptoms that the DSM-5 lists are increased phencyclidine use over longer than intended periods of time, cravings, or a strong desire to use phencyclidine and tolerance, in addition to several others [2]. The DSM-5 further states that “in individuals with phencyclidine use disorder, there may be physical evidence of injuries from accidents, fights, and falls” and that “chronic use of phencyclidine may lead to deficits in memory, speech, and cognition that may last for months” [2].

Similarly, the DSM-5 defines hallucinogen use disorder as “a problematic pattern of hallucinogen (other than phencyclidine) use leading to clinically significant impairment or distress, as manifested by at least two of the following, occurring within a 12-month period” [2]. The following symptoms listed include many of the same symptoms listed for PCP use disorder. A point of interest within the DSM-5, however, is the statement that “there is evidence for long-term neurotoxic effects of methylenedioxymethamphetamine (MDMA)/ecstasy use, including impairments in memory, psychological function, and neuroendocrine function; serotonin system dysfunction; and sleep disturbance; as well as adverse effects on brain microvasculature, white matter maturation, and damage to axons” [2].

It is necessary that these factors be considered when researching the potential medical effects of various hallucinogenic drugs. Without a thorough understanding of how psychedelics may influence an individual’s behavior and health, psychedelics cannot be considered safe for medical use. NIDA states that it conducts (and supports) research “to better understand how often and to what extent people experience tolerance, withdrawal, and other substance use disorder symptoms related to the use of psychedelic and dissociative drugs”, though minimal information has been published as of today [1].

### 4.3. Asserted Effects of Psychedelics for Medical Treatment

Psychedelics have been studied for possible use in treating a variety of mental disorders in addition to substance use disorders [52,53,54]. These studies typically are focused on serotonergic psychedelics. Serotonergic psychedelics include substances such as LSD, dimethyltryptamine (DMT), and psilocybin [1]. They are theorized to possess a very similar structure to serotonin and have an affinity for serotonin receptors [1]. However, their mechanisms and pathways are not completely understood, as serotonin is not specific to any particular function. Current psychedelic research asserts that psychedelics have the ability to “... exert therapeutic effects for psychiatric disorders by acutely destabilizing local brain network hubs and global network connectivity via amplification of neuronal avalanches, providing the occasion for brain network “resetting” after the acute effects have resolved” [55]. However, while claims in current research appear promising, many studies surrounding psychedelic use as a treatment for psychiatric issues are not long-term studies and use small sample sizes [5].

In order to consider psychedelics safe for medicinal use, research must also consider the interaction between psychedelics and prescription drugs. In cases where the prescription medication affects levels of serotonin, the use of psychedelics on top of these medications may lead to serotonin toxicity [1]. Serotonin toxicity occurs when there is too much serotonin in the brain, and it can cause mild symptoms, such as shivering and diarrhea, as well as more serious symptoms, including muscle rigidity, fever, and seizures [1]. However, it is important to note that these reported cases are rare, and further research is needed on the topic of psychedelic usage potentially causing serotonin toxicity.

### 4.4. HPPD, Heart Valve Disease, and Long QT Syndrome

Though it is rare, the use of some psychedelic drugs may cause hallucinogen persisting perception disorder (HPPD) [1]. Individuals suffering from HPPD report “flashbacks” or “perceiving the same images or scenes”, as well as the same mood changes they underwent during their experience with psychedelic drugs [1]. These experiences are typically brief, usually only lasting a few seconds or minutes, and typically occur within a week of the substance use [1]. However, many individuals suffering from HPPD have reported more severe experiences, with flashback episodes and distress continuing for longer periods and even recurring years after their initial experience with the drug [1]. A notable issue with the information regarding HPPD and its effects is that much of the research surrounding this disorder relies on self-reported information.

Additionally, the research surrounding this disorder is very limited. In addition to HPPD, some psychedelics have been associated with long-term health issues, such as ketamine-induced uropathy (a treatable yet serious condition) and mild-to-moderate heart valve disease (where the valves supporting heart function work improperly) [1]. Psychedelic usage may also be connected to long QT syndrome, which results in irregular heart function [1]. This was shown through an analysis of studies conducted between 2015 and 2020. This analysis found that ibogaine (found in the root bark of the Iboga shrub) was associated with long QT syndrome [1].

## 5. Psychedelics and Pharmaceutical Companies

Experts in the field of psychology believe that a sort of “bubble” is forming in terms of excitement around research centering on psychedelics. The medical community is concerned that the media and industry-driven “hype” surrounding these drugs will lead to public disappointment that psychedelics are not “miracle cures” for depression, PTSD, or other mental disorders. The medical community, instead, advocates for rational, steady research on psychedelics rather than “jumping the gun”, so to speak, about these drugs. Through meticulous research, information about the dangerous effects of psychedelics can be understood in order to avoid dangerous situations. Most studies centering on psychedelics, however, require extensive funding to complete. Reports now show that investment in psychedelic research has fallen to half of what it was last year. Despite this lack of investment, there is some burgeoning interest among pharmaceutical companies [5].

Pharmaceutical companies often allocate funds to researchers, universities, and private institutions (typically via grants) in order to increase research on a topic of interest. Pharmaceutical companies, however, are concerned with profit over ethical or well-constructed research. Rather, their main reason for funding various research endeavors is financial benefit. This has been consistently shown in a variety of ways, including misrepresentation of information [56]. Pharmaceutical companies continually prove that they put financial gains over the interest of public health, whether it is achieved by “marketing drugs in a variety of unethical ways intended to increase sales including falsifying research findings, deceptively advertising drugs”, or “paying physicians to write prescriptions” [24]. Additionally, pharmaceutical companies are aware that addictive drugs are best-sellers. This has been demonstrated by Purdue Pharma during the opioid epidemic as well as by various tobacco companies [57,58]. It is important to realize that pharmaceutical involvement is likely a large reason why adverse effects of psychedelic use are underreported in the current research literature.

Van Elk and Fried note that previous studies have established the risks of conflicts of interest demonstrated by researchers [5]. In fact, Van Elk and Fried state that “previous research has found that researchers with a conflict of interest are five times more likely to find a positive effect for a drug than researchers without a conflict of interest” [5]. While this fact is concerning, there are multiple ways to decrease the risk of bias. Some suggestions from Van Elk and Fried include “independent experts as researchers in every part of the study design and conduct as well as having journal reviewers be independent of the industry” [5]. Van Elk and Fried also discuss the importance of independent funding to help mitigate the risk of bias [5].

## 6. Psychedelic Research and Gaps in the Current Medical Literature

Medical research surrounding psychedelic use as a treatment for mental disorders has resulted in optimism from many researchers and medical professionals. However, it is unfortunately not uncommon for psychedelic research to present biased information due to questionable research practices. Several studies have been identified as having regularly presented misleading information that directly contradicts their data. Michiel van Elk and Fried published a review in which they address common problems within psychedelic research (as well as recommendations for remedying these issues) [5]. Within their review, it is acknowledged that there have been many other criticisms of issues within psychedelic research and that it is necessary for these issues to be addressed in future research [59,60,61,62]. Without considering these issues, psychedelic research cannot be considered reliable or safe.

### 6.1. Ketamine for the Acute Treatment of Severe Suicidal Ideation: Double-Blind, Randomised Placebo-Controlled Trial: Abbar et al. [63]

A study completed by Abbar et al. asserted in their abstract that ketamine had “persistent benefits for acute case in suicidal patients” [63]. The objective of this study was to “confirm the rapid onset anti-suicidal benefits of ketamine in the short term and at six weeks, overall and according to diagnostic group” [63]. This study was conducted in seven French teaching hospitals and included participants older than 18 years of age with current suicidal ideation. Each of these patients was voluntarily admitted to the hospital. Participants with comorbid conditions (including schizophrenia and other psychotic disorders, substance dependence, etc.) were not included in this study. There were a total of 156 participants in this study. Eighty-three of these patients were in the placebo-receiving group, while the remaining 73 received a dosage of ketamine.

Their study states that “the primary outcome was the rate of patients in full suicidal remission at day 3” [63]. However, their randomized controlled trial found that there was “no persistent benefit of ketamine over placebo at the exit timepoint of the trial in week 6” [63]. Despite this, Abbar et al. continued asserting that their findings indicated that “ketamine is rapid, safe in the short term, and has persistent benefits for acute care in suicidal patients” [63]. This demonstrates a lack of scientific integrity. Misleading claims that misrepresent the present data may lead to individuals underestimating the risk of psychedelic use when attempting to treat suicidality.

### 6.2. Rapid and Sustained Reductions in Current Suicidal Ideation Following Repeated Doses of Intravenous Ketamine: Secondary Analysis of an Open-Label Study: Ionescu et al. [64]

Additionally, a study completed by Ionescu et al. misrepresents data results through the title of their article. This study aimed to explore the “extent to which ketamine reduces thoughts of suicide in depressed patients with current suicidal ideation” [64]. There were 14 participants in this study, all of which were diagnosed with major depressive disorder and were “recruited for the presence of current, stable suicidal thoughts” [64]. These participants received “open-label ketamine infusions over 3 weeks” [64]. After three months, researchers followed up with participants to measure their current levels of suicidal ideation (using the Columbia Suicide Severity Rating Scale and the Suicide Item of the 28-item Hamilton Depression Rating Scale) [64].

The title “Rapid and sustained reductions in current suicidal ideation following repeated doses of intravenous ketamine: secondary analysis of an open-label study” seems positive at first glance. However, when looking at the data presented, it is shown that only 2 out of 14 patients within this study demonstrated sustained improvement after a 3-month follow-up [64]. Furthermore, this information is portrayed as positive. Ionescu et al. state that “in this preliminary study, repeated doses of open-label ketamine rapidly and robustly decreased suicidal ideation in pharmacologically treated outpatients with treatment-resistant depression with stable suicidal thoughts; this decrease was maintained or at least 3 months following the final ketamine infusion in 2 patients” [64]. It is important to recognize that less than 15% of the participants experienced a sustained decrease in suicidal ideation at the 3-month follow-up, which hardly appears to be a significant number of participants. Instead, this study only includes patients who have achieved remission during the acute treatment phase in their calculations post-follow-up (which still would not be a statistically significant percentage).

### 6.3. Rapid Antidepressant Effects of the Psychedelic Ayahuasca in Treatment-Resistant Depression: A Randomized Placebo-Controlled Trial: Palhano-Fontes et al. [65]

In addition to misleading reports of data, there is the issue of poorly constructed double-blind studies. As demonstrated through the Good Friday Experiment, it is essential that researchers preserve the integrity of a double-blind study [65]. Additionally, it is extremely important that studies detail any issues with the preservation of their double-blind study within their published articles. Palhano-Fontes et al. conducted a double-blind study with the goal of testing “the antidepressant effects of ayahuasca” [65]. To do so, Palhano-Fontes et al. conducted a “parallel-arm, double-blind, randomized placebo-controlled trial” that included 29 patients with treatment-resistant depression. These patients were assigned to receive either a dosage of ayahuasca or a placebo [65].

The effect of the ayahuasca dosage on the participants’ depression was measured by the Montgomery–Åsberg Depression Rating Scale (MADRS) as well as the Hamilton Depression Rating Scale. These measurements were taken at baseline, 1, 2, and 7 days after dosing. During this study, 14 individuals received the psychedelic, and 15 individuals received a placebo [65]. Palhano-Fontes et al. reported that “blindness was adequately preserved” throughout their study when, unfortunately, it had not been [65]. Each member of the treatment group maintained that they had received ayahuasca. However, less than half of the members of the placebo group believed the same [65]. Therefore, it is impossible for blindness to have been preserved.

### 6.4. Increased Global Integration in the Brain After Psilocybin Therapy for Depression: Daws et al. [66]

Michiel van Elk and Fried also discuss a major issue within a study completed by Daws et al. in which two treatment arms are compared [66]. Their study uses psilocybin-assisted therapy and ultimately concludes that one treatment “outperformed the other despite the lack of a statistically significant interaction term between the treatments” [66]. In this study, patients with treatment-resistant depression were administered two doses of psilocybin 7 days apart (10 mg and 25 mg.) A second trial was also completed after this, during which patients received either 2 × 25 mg oral psilocybin, 3 weeks apart, plus 6 weeks of daily placebo (‘psilocybin arm’) or 2 × 1 mg oral psilocybin, 3 weeks apart, plus 6 weeks of daily escitalopram (10–20 mg) (‘escitalopram arm’) [66]. Without accurate reports of a lack of significance, this study cannot be considered scientifically valid. In fact, an underreporting of null hypotheses and lack of significant difference only serves to negate positive claims of psychedelics’ proposed therapeutic effects. It is unknown whether this misrepresentation of data was a result of publication bias or financial pressure, but regardless, it is unsafe to promote inaccurate information to the public.

### 6.5. Single-Dose Psilocybin Treatment for Major Depressive Disorder: A Randomized Clinical Trial: Raison et al. [67]

Raison et al. completed their single-dose psilocybin study with the goal of examining the efficacy and safety of psilocybin when treating patients with major depressive disorder [67]. This was studied in a “randomized, placebo-controlled, 6-week trial” with 104 adult participants [67]. These participants were randomized in a “1:1 ratio to receive a single dose of psilocybin versus niacin placebo”, which was administered with psychological support. The dosage of psilocybin was 25 mg, and exclusion criteria for this study included “history of psychosis or mania, active substance use disorder, and active suicidal ideation with intent”. This study concluded that a 25 mg dose of psilocybin (accompanied by psychological support) was associated with a rapid and sustained antidepressant effect [67].

There are several criticisms that arise when examining this study. Most notably, there is the question of what viability a single-dose study has when examining psychedelics as a treatment for both PTSD and depression. Additionally, the majority of psychedelic studies encompass a therapeutic component. Despite this, there has been minimal information regarding whether the noted treatment of symptoms is actually due to the psilocybin or rather is an effect of therapeutic treatment. In fact, the National Institute of Mental Health explains that psychotherapy is already a viable and evidence-based treatment for depression [68]. Therefore, it is necessary that studies examining the effects of psychedelics using therapeutic strategies look into the potential that the positive effects are potentially not caused by psychedelics but rather by evidence-based therapeutic treatments.

### 6.6. Absence of Efficacy Research and Data on Long-Term Effects

NIDA itself states that adverse effects are likely being underreported [1]. This is a massive issue, especially when attempting to understand the safety of psychedelic use. It is unknown whether the underreporting of adverse effects is due to personal bias, financial gains, or any other motivating factors. Regardless, this lack of information has the potential to leave individuals who are interested in psychedelic use for medical benefit open to a multitude of unexpected effects.

The FDA requires that “evidence that the drug will benefit the target population should outweigh any risks and uncertainties” as part of the drug approval process [19]. However, this requirement has not been adequately upheld. In fact, when trying to determine whether esketamine was a valid and safe treatment for depression, the FDA demonstrated that minimal research is needed [69,70]. Esketamine failed five out of six initial efficacy trials. Additionally, the sixth trial only passed statistically and not clinically [69]. Despite this, the FDA failed to maintain its previous guidelines and considered this sufficient research. With that in mind, it is necessary that researchers conducting studies on psychedelics publish each adverse effect (including addictive consequences) that arises within their study. Without transparency on the potential outcomes of psychedelic use, it cannot be considered safe or approved by the FDA.

It may also be worth researching whether or not psychedelics are truly affecting mood-related disorders or simply causing an intoxicated state. As an example, alcohol has been shown to cause a temporary state of happiness (although it does not increase overall life satisfaction in the long term). Currently, there are some studies showing that psychedelics have a much longer effect on an individual’s mental state; however, it may be beneficial to conduct additional longitudinal studies in order to solidify this claim [71]. Alcohol has been shown to have negative long-term effects despite momentary feelings of happiness, and it is necessary to determine that psychedelics do not have a similar effect. In general, the research literature surrounding the long-term mental and physical effects of psychedelic use is severely lacking. While the current claims in the literature appear optimistic, it is necessary to conduct further efficacy research in order to truly understand the effects of psychedelics prior to determining them as safe for medical use.

## 7. FDA’s Decision on MDMA Rescheduling

MDMA (also known as ecstasy or molly) is “a lab-made (synthetic) drug that has effects similar to stimulants like methamphetamine” [72]. It recently gained traction in the media as a potential psychotherapeutic treatment for individuals suffering from severe PTSD. Lykos Therapeutics, an organization established by MAPS (Multidisciplinary Association for Psychedelic Studies), has been an outspoken proponent of MDMA as a medicinal tool. However, on 9 August 2024, Lykos Therapeutics was informed by the FDA that the agency had decided not to approve MDMA-assisted therapy for PTSD at the present time [73]. This decision stated that Lykos would need to provide further studies on the safety and efficacy of the treatment, specifically requesting an additional late-stage clinical trial to further study MDMA’s safety and efficacy [74,75]. Lykos, however, hopes to ask the FDA to reconsider their decision [73]. One issue that was raised by the FDA was the “inability to distinguish MDMA from a placebo effect” due to many patients correctly identifying whether they have received a placebo or drug (which has consistently been shown to be a concern within psychedelic research) [75].

### MAPS and Organizational Errors

One of the largest proponents of MDMA for medicinal use is the Multidisciplinary Association for Psychedelic Studies (MAPS). In past years, MAPS has conducted a variety of studies attempting to establish the therapeutic properties of psychedelic drugs. However, MAPS gained media attention when a former patient in a MAPS study publicly detailed her experience with the organization, explaining that she was assaulted during their study. Video footage has since been released that shows two B.C. therapists “cuddling, spooning, blindfolding, and pinning down a distressed PTSD patient” while conducting their MDMA study [76]. This prompted a review by MAPS regarding safety practices and policies, during which MAPS maintained that they were unaware of any signs of ethical violation during their study [76]. However, MAPS spokesperson Betty Aldworth later stated that the “organization’s staff did not actually view the videos until November 2021, six years after they were filmed” [76]. A smaller (yet still notable) point of concern arises when examining one of the B.C. therapists involved in the trial. This therapist was previously listed as a psychologist on MAPS’ website; however, he was not licensed during the time of that study [77]. In fact, his license had lapsed four years prior.

The study completed by MAPS is a clear example of how a lack of safety practices not only hinders progress within psychedelic research but also can be incredibly unsafe. Situations like what occurred within these MDMA trials are not unique in nature and speak to the credibility of these organizations. The purpose of discussing safety concerns within these MDMA studies is not to discourage future psychedelic research or disparage existing organizations but rather to emphasize the importance of the safety and efficacy of psychedelics when considering them for medicinal use. This claim is not independent of psychedelics but, in fact, applies to all drugs that aim to be rescheduled. Ultimately, scientific integrity and thorough research are necessary when examining these drugs’ effects on public health.

## 8. Discussion

Various studies have been conducted to examine if psychedelics can be used to treat a variety of mental disorders and substance use disorders [52,53,54]. However, this is still an emerging area of research. As demonstrated by the FDA decision regarding MDMA, an extensive amount of clinical research is necessary to determine the safety and efficacy of these drugs. The FDA decided that further studies would be necessary and specifically requested an additional late-stage clinical trial [74,75]. While this may be a costly study to produce, it has been deemed necessary to ensure the safety of the public should the drug be accepted for therapeutic use.

For psychedelics to be considered for medical or therapeutic use, further studies may be required. While interest has been increasing surrounding the use of psychedelics, it is necessary to determine the effects these drugs may have on public health. This is not unique to psychedelics. Rather, all drugs scheduled under the Controlled Substance Act must provide extensive research demonstrating their safety and medical uses to be rescheduled.

It is necessary to recognize that psychedelics may affect individuals in widely varying ways. As previously discussed, an individual’s age, unique biology, sex, personality, history of drug use, mood, expectations, mindset, and surroundings all may impact an individual’s unique experience with psychedelics [1]. Therefore, to fully understand the scope of adverse effects psychedelics may have on an individual, research must consider each of these factors thoroughly.

## 9. Conclusions

The safety and efficacy of psychedelics are still highly understudied and doubtful. It is necessary to consider the history of psychedelic abuse and construct research with a high level of diligence. Additionally, psychedelics have a high chance of worsening pre-existing mental conditions and effects (such as increasing levels of anxiety in individuals already suffering from anxiety disorders) [78]. It is necessary that researchers study these effects in great detail in order to fully understand the adverse effects of psychedelic use prior to their potential medical use. It is also important to note the role that pharmaceutical companies have in psychedelic research and be aware of the bias that may arise from such involvement. Many current studies of psychedelic drugs employ methodologies that are not standardized, have small sample sizes, have poorly constructed double-blind procedures, and merely look at statistical analyses that are not fit for drawing accurate conclusions as to the drug’s medicinal qualities [5]. Caution must be taken going forward in studying the safety of psychedelic drugs prior to any implementation within the medical field.

## Data Availability

No new data were created or analyzed in this study.

## Abbreviations

BC	British Columbia
CSA	Controlled Substance Act
DMT	Dimethyltryptamine
DSM-5	*The Diagnostic and Statistical Manual of Mental Disorders, Fifth Edition*
HPPD	Hallucinogen Persisting Perception Disorder
FDA	United States Food and Drug Administration
LSD	Lysergic Acid Diethylamide
MADRS	Montgomery–Åsberg Depression Rating Scale
MAPS	Multidisciplinary Association for Psychedelic Studies
MDMA	3,4-methylenedioxymethamphetamine
NIDA	National Institute on Drug Abuse
PCP	Phencyclidine
PTSD	Post-Traumatic Stress Disorder
QT	QT Interval (a measurement of the electrical activity in the heart’s lower chambers between the Q and T waves on an electrocardiogram)
